# The Influence of Anthropometric, Kinematic and Energetic Variables and Gender on Swimming Performance in Youth Athletes

**DOI:** 10.2478/hukin-2013-0083

**Published:** 2013-12-31

**Authors:** Jorge E Morais, Nuno D Garrido, Mário C Marques, António J Silva, Daniel A Marinho, Tiago M Barbosa

**Affiliations:** 1 Department of Sport Sciences, Polytechnic Institute of Bragança, Bragança, Portugal.; 2 Department of Sport Sciences, University of Trás-os-Montes and Alto Douro, Vila Real, Portugal.; 3 Department of Sport Sciences, University of Beira Interior, Covilhã, Portugal.; 4 National Institute of Education, Nanyang Technological University, Singapore.; 5 Research Centre in Sports, Health and Human Development, Vila Real, Portugal.

**Keywords:** swimming, performance, skill, cohort comparison

## Abstract

The aim of this study was to assess the: (i) gender; (ii) performance and; (iii) gender versus performance interactions in young swimmers’ anthropometric, kinematic and energetic variables. One hundred and thirty six young swimmers (62 boys: 12.76 ± 0.72 years old at Tanner stages 1–2 by self-evaluation; and 64 girls: 11.89 ± 0.93 years old at Tanner stages 1–2 by self-evaluation) were evaluated. Performance, anthropometrics, kinematics and energetic variables were selected. There was a non-significant gender effect on performance, body mass, height, arm span, trunk transverse surface area, stroke length, speed fluctuation, swimming velocity, propulsive efficiency, stroke index and critical velocity. A significant gender effect was found for foot surface area, hand surface area and stroke frequency. A significant sports level effect was verified for all variables, except for stroke frequency, speed fluctuation and propulsive efficiency. Overall, swimmers in quartile 1 (the ones with highest sports level) had higher anthropometric dimensions, better stroke mechanics and energetics. These traits decrease consistently throughout following quartiles up to the fourth one (i.e. swimmers with the lowest sports level). There was a non-significant interaction between gender and sports level for all variables. Our main conclusions were as follows: (i) there are non-significant differences in performance, anthropometrics, kinematics and energetics between boys and girls; (ii) swimmers with best performance are taller, have higher surface areas and better stroke mechanics; (iii) there are non-significant interactions between sports level and gender for anthropometrics, kinematics and energetics.

## Introduction

Technical training (Barbosa et al., 2010a) and endurance training (Marinho et al., 2009) are two major determinants of young swimmers’ performance. Young swimmers’ training and competition should be monitored on a regular basis in order to design appropriate training sets and enhance performance (Marinho et al., 2011) as currently occurs in adult/elite counterparts (Komar et al., 2012).

It seems that anthropometrics are highly related with young swimmers’ performance. For instance, arm span (AS) is one of the best performance predictors (Lätt et al., 2010), while positive correlations between hand and foot size with performance also exist (Helmuth, 1980). Regarding stroke mechanics, stroke index (SI) was the variable having the highest association with performance (Vitor and Böhme, 2010; Lätt et al., 2009). Besides anthropometrics and technical abilities, energetics play a significant role in young swimmers’ performance. Critical velocity (CV) was highly correlated with young swimmers’ performance (Vitor and Böhme, 2010). Also reported was an association between aerobic capacity and performance (Barbosa et al., 2010a).

Regarding the gender gap, pubescent and adult/elite male and female swimmers differ in measures of anthropometrics, kinematics and energetics. In young swimmers’ gender differences were reported in body fat (Zuniga et al., 2011). Even so, it seems that there are non-significant differences in the anthropometrics, growth and maturation processes between both genders at this early age (Malina et al., 2004). Body mass (BM), height (H) and several body circumferences and areas presented non-significant differences between boys and girls (Zuniga et al., 2011). In the kinematics domain, at least one paper (Bielec et al., 2008) reported significant differences between boys and girls for swimming velocity (v) and stroke frequency (SF). Concerning energetics, total energy expenditure and energy cost of a given swimming velocity are commonly evaluated in adult/elite swimmers (Barbosa et al., 2010b). Nevertheless, young swimmers’ energetic profile (e.g. aerobic capacity) assessment should be straightforward and noninvasive. Hence, the estimation of some of those parameters is necessity (Barbosa et al., 2010a). Regarding aerobic capacity, Greco and Denadai (2005) also found non-significant differences between genders. However, in these studies, it was not stated whether they were comparing boys and girls of the same sports level. In this sense, it is of major interest to dispose of hypothetical differences between boys and girls that compete at the same level, instead of pooling into the same gender group subjects at different sports levels.

There are few studies describing and analyzing in a single paper the effect of anthropometrics, kinematics and energetics on young swimmer’s performance. The few exceptions are Barbosa et al. (2010a), Vitor and Böhme (2010), Lätt et al. (2010), Jürimäe et al. (2007), and Greco et al. (2007). In addition, the sample sizes of most studies can be considered as small to moderate, leading to concerns regarding data statistical power.

This study intended to analyze the: (i) gender effect on anthropometrics, kinematics and energetics; (ii) sports level effect on anthropometrics, kinematics and energetics and; (iii) the sports level-gender interactions on anthropometrics, kinematics and energetics.

## Material and Methods

### Participants

The sample was composed of 136 young swimmers (62 boys: 12.76 ± 0.72 years old at Tanner stages 1–2 by self-evaluation; and 64 girls: 11.89 ± 0.93 years old at Tanner stages 1–2 by self-evaluation; 24.66 ± 2.91 training sessions per month) participating regularly in national and regional level competitions.

Coaches and parents gave their consent for swimmers’ participation in this study and all procedures accorded with the Helsinki Declaration regarding Human Research. The University of Trás-os-Montes and Alto Douro Ethic Committee also approved the study design (ethic review: UTAD-2011-219).

#### Performance data collection

Swimming performance was assessed by time lists of the 100 m freestyle event in short course competitions (25 m swimming pool) at local, regional or national level competitions. For each gender, performance data was used to divide swimmers into quartiles (i.e. cohort groups) according to their sports level. Further effects and interaction assessments were made according to the particular group (sports level). Quartile one represents the highest performance level and quartile four the lowest. The time gap between all evaluations and swimming performance variables was less than two weeks (Barbosa et al., 2010a).

#### Anthropometric data collection

In the anthropometric domain data were collected three times: (i) BM; (ii) H; (iii) AS; (iv) trunk transverse surface area (TTSA); (v) hand surface area (HSA) and; (vi) foot surface area (FSA). The average value of the three trials was used for further analysis. Body mass was measured with the subjects in the upright position with a digital scale (SECA, 884, Hamburg, Germany). The H was measured in the anthropometrical position from vertex to the floor with a digital stadiometer (SECA, 242, Hamburg, Germany). Arm span was measured in the upright and orthostatic position with lateral abduction of both arms at a 90º angle with the trunk. Both arms and fingers were fully extended and the distance between the tip of each third finger was measured with a flexible anthropometric tape (RossCraft, Canada) (ICC = 0.99).

Surface areas (i.e. TTSA, HSA and FSA) were computed by digital photogrammetry. For TTSA assessment, subjects were photographed with a digital camera (DSC-T7, Sony, Tokyo, Japan) in the transverse plane from above (Caspersen et al., 2010). Subjects were on land in the upright position simulating the hydrodynamic position (i.e. arms fully extended above the head, one hand above the other with fingers also fully extended). Subjects were placed near a 0.945 m length 2D calibration frame wearing a regular textile swimsuit, cap and goggles (Morais et al., 2011) (ICC = 0.97).

For the HSA measurement, swimmers put their dominant hand with fingers in position for normal swimming near to a 2D calibration frame on the scan surface of a copy machine (Xerox 4110, Norwalk, Connecticut, USA) (Morais et al., 2011). Thereafter, the perimeter of the HSA was digitized in the Xerox machine and files were converted in *.pdf format. For the FSA measurement the procedure was the same. Afterwards, both surface areas were computed with specific software (Universal Desktop Ruler, v3.3.3268, AVPSoft, USA) (ICC = 0.99).

#### Kinematic data collection

Swimming velocity, speed fluctuation (dv), stroke length (SL) and SF were selected as kinematic variables. Each swimmer made three 25-m freestyle trials with an underwater start. Average value of the three trials was used for further analysis. Swimmers performed the trials alone without other swimmers in the same swim lane to reduce drafting and/or pacing effects and were advised to reduce gliding after the start (Barbosa et al., 2010a).

A speedometer cable (Swim speedometer, Swimsportec, Hildesheim, Germany) was attached to the swimmers’ hip. Data was acquired on-line at a sampling rate of 50 Hz, exported to a signal processing software (AcqKnowledge v. 3.5, Biopac Systems, Santa Barbara, USA) and filtered with a 5 Hz cutoff low-pass 4^th^ order Butterworth filter (Barbosa et al., 2013). Swimming velocity was measured in the middle 15-m as:

(1) $$v=\frac{d}{t}$$

Where *v* is the mean swimming velocity (in m·s^−1^), *d* is the distance covered (in m) and *t* is the time spent to cover that distance (in s). Speed fluctuation was computed as (Barbosa et al., 2008):

(2) $$\mathrm{dv}=\frac{\sqrt{\Sigma_{t}{\left(v_{t}-\sigma \right)}^{2}F_{t}/n}}{\Sigma_{t}v_{t}F_{t}/n}$$

Where *dv* represents speed fluctuation (dimensionless), *v* represents the mean swimming velocity (in m·s^−1^), *v_i_* represents the instant swimming velocity (in m·s^−1^), *F_i_* represents the absolute frequency and *n* represents the number of observations. Stroke length was computed as (Craig and Pendergast, 1979):

(3) $$\mathrm{SL}=\frac{v}{\mathrm{SF}}$$

Where *SL* represents stroke length (in m), *v* represents the mean swimming velocity (in m·s^−1^) and *SF* represents the stroke frequency (in Hz). Stroke frequency was measured with a chrono-frequency counter during three consecutive strokes by two expert evaluators (ICC = 0.98)

#### Energetic data collection

In the energetic domain the SI, propulsive efficiency (η_p_) and CV were selected. The SI was computed as (Costill et al., 1985):

(4) SI=SL⋅v

Where *SI* represents stroke index (in m^2^·s^−1^), *SL* represents stroke length (in m) and *v* is the mean swimming velocity (in m·s^−1^). The η^p^ was computed as (Zamparo et al., 2005):

(5) $$\eta_{p}=\left[\left(\frac{v\cdot 0.9}{2\pi \cdot \mathrm{SF}\cdot l}\right)\cdot \frac{2}{\pi }\right]\cdot 100$$

Where *η_p_* represents propulsive efficiency (in %), *v* represents the swimming velocity (in m·s^−1^), *SF* represents the stroke frequency (in Hz) and *l* represents the arm length (in m). The CV was assessed based on the 200 m and 800 m freestyle short course events (Barbosa et al., 2010a). It was computed using the slope of the simple linear regression model, according to Wakayoshi et al. (1992):

(6) d=a⋅t+b

where *d* represents the distance of the swim event (in m), *a* represents the slope of the fit line, *t* represents the time spent to cover the distance (in s), and *b* represents the y interception in the origin of the xx axis.

#### Statistical analysis

The Kolmogorov-Smirnov and Levene tests were used to analyze normality and homocedasticity assumptions, respectively. The total sample was divided into quartiles according to best performance time in 100-m freestyle events. Descriptive statistics (mean ± 1SD) were computed for each quartile and gender for the anthropometric, kinematic and energetic variables selected.

Data variation was analyzed using a twoway ANOVA (gender versus sports level) followed by the post-hoc test of Bonferroni to verify differences between quartiles (p ≤ 0.05). Also computed was the size effect based on the eta-squared (η^2^), and interpreted as (Ferguson, 2009): (i) without effect if 0 < η^2^ < 0.04; (ii) minimum if 0.04 < η^2^ < 0.25; (iii) moderate if 0.25 < η^2^ < 0.64 and; (iv) strong if η^2^ > 0.64.

## Results

**Figure 1:**
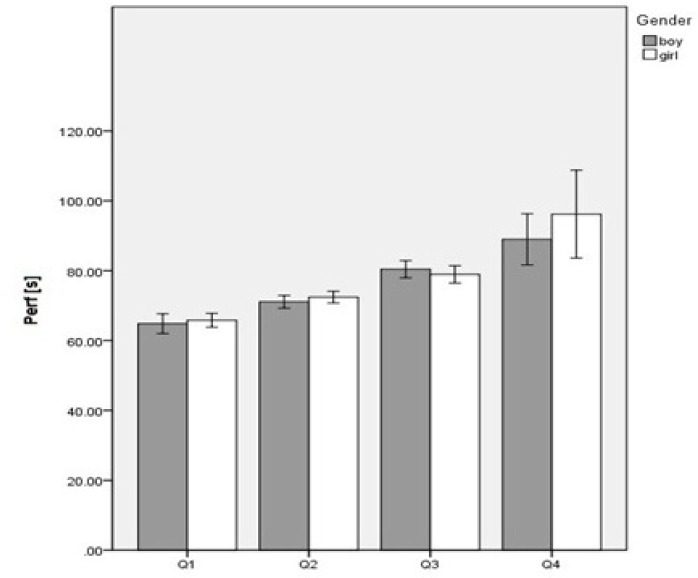
Mean data comparison by sports level, and by sports level according to gender of swimming performance. Perf – performance

Figure 1 presents the performance variation by sports level and by performance level according to gender. There was a non-significant gender effect on performance. Performance was higher (i.e. less time spent to cover the distance) in quartile 1, and decreased up to quartile 4 (p < 0.001; 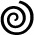
2 = 0.76). The interaction between gender and sports level was non-significant, very close to the cut-off value of the null hypothesis rejection area (p < 0.05), and with a strong effect (p = 0.06; 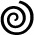
2 = 0.78).

**Figure 2:**
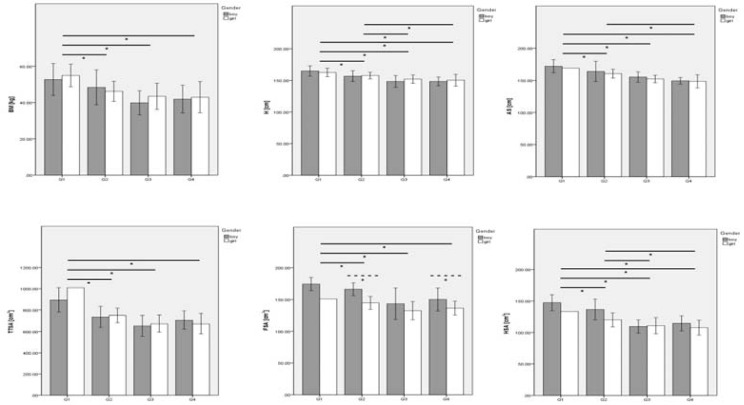
Mean data comparison by sports level, and by sports level according to gender of anthropometric selected variables. BM – body mass; H – height; AS – arm span; TTSA – trunk transverse surface area; FSA – foot’s surface area; HAS – hand’s surface area. Solid lines represent p ≤ 0.05 for the sports level effect; dash lines represent p ≤ 0.05 for gender effect

Figure 2 presents the anthropometric variations based on sports level and interaction between gender and performance level. Gender had a non-significant effect on BM, H, AS, TTSA and HSA. However, for FSA there was a significant and moderate gender effect (p = 0.001; 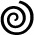
2 = 0.35). FSA was higher for boys than girls in quartile 2 (p = 0.01) and quartile 4 (p = 0.05).

The sports level had a significant and moderate effect on all anthropometric variables such as BM (p < 0.001; 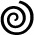
2 = 0.24), H (p < 0.001; 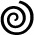
2 = 0.37), AS (p < 0.001; 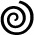
2 = 0.55), TTSA (p < 0.001; 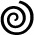
2 = 0.50), FSA (p = 0.005; 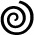
2 = 0.47) and HSA (p < 0.001; 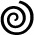
2 = 0.59). Quartile 1 had a higher BM, TTSA and FSA, followed by quartile 2, 4 and 3, respectively. H, AS and HSA decreased from quartile 1 to quartile 4. The interaction between gender and the sports level was non-significant for all selected variables, but with a moderate-strong effect.

**Figure 3:**
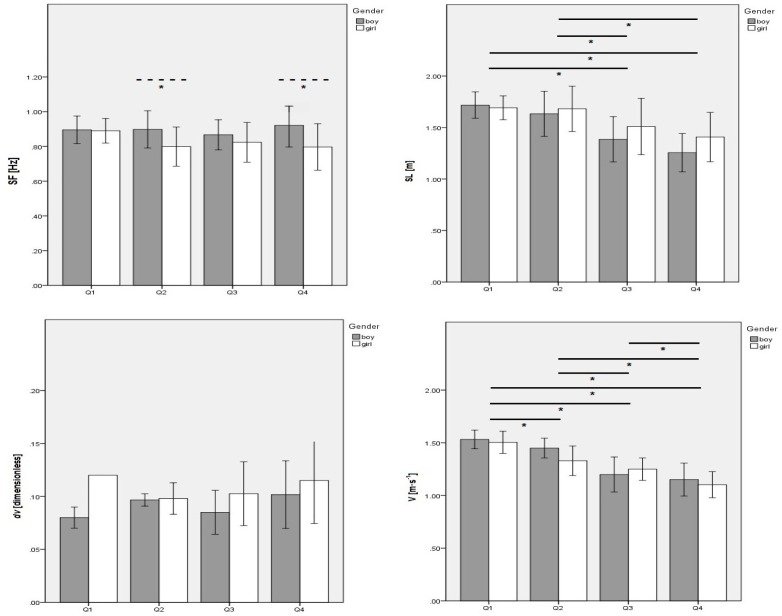
Mean data comparison by sports level, and by sports level according to gender of kinematic selected variables. SF – stroke frequency; SL – stroke length; dv – speed fluctuation; V – swimming velocity. Solid lines represent p ≤ 0.05 for the sports level effect, dash lines represent p ≤ 0.05 for gender effect

Figure 3 presents the kinematic variations based on the sports level and the interaction between gender and the performance level. Gender had a significant but minimum effect on the SF (p = 0.003; 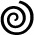
2 = 0.12). Remaining variables showed non-significant effects (i.e. SL; dv and; v). SF was higher in boys than girls in quartile 2 (p = 0.02) and quartile 4 (p = 0.04). The sports level did not have a significant effect on the SF and dv, but did so on the SL (p < 0.001; 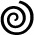
2 = 0.32) and v (p < 0.001; 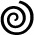
2 = 0.64). Both SL and v decreased from quartile 1 to quartile 4. The interaction between gender and the sports level was non-significant for all kinematic variables and a moderate-strong effect was observed.

**Figure 4:**
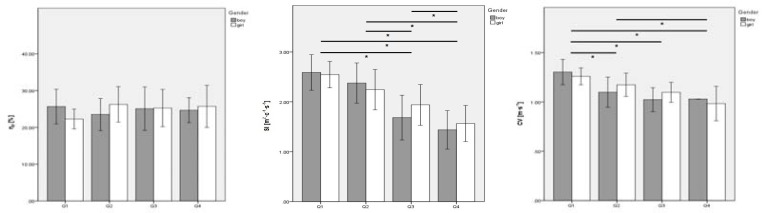
Mean data comparison by sports level, and by sports level according to gender of energetic selected variables. η_p_ – propulsive efficiency; SI – stroke index; CV – critical velocity. Solid lines represent p ≤ 0.05 for the sports level effect

Figure 4 presents the energetic variations based on the sports level and interaction between gender and the sports level. There was a non-significant gender effect on the 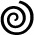
p, SI and CV. The sports level did not have a significant effect on 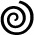
p, but did so on SI (p < 0.001; 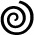
2 = 0.55) and CV (p < 0.001; 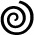
2 = 0.46). Stroke index and CV were higher in quartile 1, and decreased up to quartile 4. The interaction between gender and the sports level was non-significant for 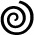
p, SI and CV.

## Discussion

The aim of this study was to analyze the gender and sports level effects on young swimmers’ anthropometrics, kinematics and energetics. Overall the data showed neither a gender effect nor gender versus sports level interaction. However, a sports level effect was reported. Present data shows that there is no gender gap comparing swimmers with the same sports level. Although the high-level swimmers significantly differ from lower skilled ones. Faster swimmers are taller, with higher AS and surface areas, higher SL, v, SI and CV. These features are positively related to young swimmers’ performance (Jürimäe et al., 2007; Vitor and Böhme, 2010).

Research with young swimmers is not as common as for adult/elite counterparts (e.g. Schnitzler et al., 2011). Literature clearly reports the anthropometric, kinematic and energetic influence on adult/elite swimmers’ performance. Such relationships are not so evident in their younger counterparts. Moreover, most of the youth swimming research has used smaller sample sizes than this one. Previous cohort designs, notably comparing the gender effect, failed to record whether swimmers in different sub-groups were of the same sports level. Selected variables are reported on a regular basis in young swimmer’s literature (Barbosa et al., 2010a; Lätt et al., 2010; Vitor and Böhme, 2010; Silva et al., 2007; Jürimäe et al., 2007). These variables are informative and useful for coaches in helping them to conduct an evidence-based practice.

Swimmers in quartile one (i.e. high sports level) were heavier, taller, with higher AS and surface areas in comparison with the remaining groups. The same trend occurred in the stroke mechanics and energetic variables. High level swimmers had higher values for these variables (kinematics and energetics) in comparison with remaining quartiles. Thus anthropometrics, kinematics and energetics had a significant effect on performance, particularly between the group of high-level swimmers and the remaining ones. Literature reports that anthropometrics, such as H, AS (e.g. Jurimäe et al., 2007) and surface areas (e.g. Helmuth, 1980) are related to a better performance in young swimmers. It is known that v improvement depends on increased SL (Tella et al., 2002). The SI and CV were the best predictors of swimming performance in young swimmers (Vitor and Böhme, 2010). However, to date no study has assessed all these domains in a single and large sample size as reported in this paper.

The new trends in swimming research suggest a deterministic relationship, where several scientific domains are related with each other and may have a direct or indirect influence on performance. Swimming performance is determined by the swimmer’s energetic profile, and this last one is influenced by his/her biomechanical behavior (Barbosa et al., 2010b). On the other hand, biomechanics are determined by motor control and anthropometrics (Barbosa, 2011). However, these models were developed for adult/elite swimmers, which can be considered as a main limitation when applied to young counterparts. Indeed, experimental data on this topic with young swimmers is scarce. A few research projects have been conducted by Tella et al. (2002), Jürimäe et al. (2007) and Barbosa et al. (2010a). In these, an increased AS imposed an increase in the SL; while SL increased the v and thus enhanced performance (Tella et al., 2002). It was also suggested that a lower energy cost in young swimmers was related to differences in anthropometric variables such as H and AS (Jürimäe et al., 2007). The relationship between the kinematics and energetic domains with performance was also studied through confirmatory research designs. Performance was linked to η_p_, CV (i.e. energetics) and v (i.e. kinematics) and this latter was dependent on SL and SF (i.e. kinematics) (Barbosa et al., 2010a). Thus, high-level swimmers are taller, and have a higher AS, allowing them to achieve a higher SL, which imposes a higher v, higher SI, higher CV and therefore, a better performance.

Two reports that differ between genders in young swimmers are related to body fat (Zuniga et al., 2011) and SF (Bielec et al., 2008). There was a gender effect for the FSA and the SF only in quartiles two and four in both variables with moderate and minimum effects, respectively. Based on the effect size rule of thumb adopted, differences seem to be quite reduced. Therefore, in stating that there are obvious FSA and SF gender differences, some caution should be taken. For the remaining anthropometric, kinematic and energetic variables there were no gender effects.

As feedback for coaches when designing young swimmers’ training sets, it should be emphasized that despite the gender the high level swimmers are characterized by higher body dimensions and also higher values of stroke mechanics and energetic variables. This matches data from deterministic models reporting the interaction between anthropometric, kinematic and energetic domains in competitive swimming (Barbosa et al., 2010a, 2010b). So, in relating results from those models with data from this research, it seems that swimmers with the highest H have higher AS and surface areas as well. These surface areas, when properly oriented, induce an increase in propulsive forces. By consequence, such swimmers can achieve a higher SL and consequently a higher v. Because they have a higher SL and v, SI increases swimmers’ ability to reach a higher CV and therefore an enhanced performance.

The main limitations of this research may be summarized as follows: (i) with regard to energetics, variables from oximetry or blood lactate were not included since research in children ought to avoid as far as possible invasive and complex procedures; (ii) regarding kinematics, more sophisticated technique evaluations such as videometry were not used since this was thought too time consuming and complex to be conducted; (iii) variables from other domains that may also play an important role in young swimmers’ performance (e.g. motor control, hydrodynamics, genetics, strength and conditioning) were not taken into consideration in the present study.

As main conclusions: (i) there are non-significant variations in performance, anthropometrics, kinematics and energetics between boys and girls; (ii) high sports level swimmers from both genders are taller, with higher AS, surface areas, SL, v, SI and CV; (iii) there are non-significant interactions between the sports level and gender for performance, anthropometrics, kinematics and energetics.
